# Methodologies for *in vitro* and *in vivo* evaluation of efficacy of antifungal and antibiofilm agents and surface coatings against fungal biofilms

**DOI:** 10.15698/mic2018.07.638

**Published:** 2018-06-14

**Authors:** Patrick Van Dijck, Jelmer Sjollema, Bruno P. A. Cammue, Katrien Lagrou, Judith Berman, Christophe d’Enfert, David R. Andes, Maiken C. Arendrup, Axel A. Brakhage, Richard Calderone, Emilia Cantón, Tom Coenye, Paul Cos, Leah E. Cowen, Mira Edgerton, Ana Espinel-Ingroff, Scott G. Filler, Mahmoud Ghannoum, Neil A.R. Gow, Hubertus Haas, Mary Ann Jabra-Rizk, Elizabeth M. Johnson, Shawn R. Lockhart, Jose L. Lopez-Ribot, Johan Maertens, Carol A. Munro, Jeniel E. Nett, Clarissa J. Nobile, Michael A. Pfaller, Gordon Ramage, Dominique Sanglard, Maurizio Sanguinetti, Isabel Spriet, Paul E. Verweij, Adilia Warris, Joost Wauters, Michael R. Yeaman, Sebastian A.J. Zaat, Karin Thevissen

**Affiliations:** 1 VIB-KU Leuven Center for Microbiology, Leuven, Belgium.; 2 KU Leuven Laboratory of Molecular Cell Biology, Leuven, Belgium.; 3 University of Groningen, University Medical Center Groningen, Department of BioMedical Engineering, Groningen, The Netherlands.; 4 Centre for Microbial and Plant Genetics, KU Leuven, Leuven, Belgium.; 5 Department of Plant Systems Biology, VIB, Ghent, Belgium.; 6 Department of Microbiology and Immunology, KU Leuven, Leuven, Belgium.; 7 Clinical Department of Laboratory Medicine and National Reference Center for Mycosis, UZ Leuven, Belgium.; 8 School of Molecular Cell Biology and Biotechnology, Faculty of Life Sciences, Tel Aviv University, Ramat Aviv, Israel.; 9 Institut Pasteur, INRA, Unité Biologie et Pathogénicité Fongiques, Paris, France.; 10 Department of Medical Microbiology and Immunology, University of Wisconsin-Madison, Madison, Wisconsin, USA.; 11 Department of Medicine, University of Wisconsin-Madison, Madison, Wisconsin, USA.; 12 Unit of Mycology, Statens Serum Institut, Copenhagen, Denmark.; 13 Department of Clinical Microbiology, Rigshospitalet, Copenhagen, Denmark.; 14 Department of Clinical Medicine, University of Copenhagen, Copenhagen, Denmark.; 15 Leibniz Institute for Natural Product Research and Infection Biology - Hans Knoell Institute (HKI), Dept. Microbiology and Molecular Biology, Friedrich Schiller University Jena, Institute of Microbiology, Jena, Germany.; 16 Department of Microbiology & Immunology, Georgetown University Medical Center, Washington DC, USA.; 17 Severe Infection Research Group: Medical Research Institute La Fe (IISLaFe), Valencia, Spain.; 18 Laboratory of Pharmaceutical Microbiology, Ghent University, Ghent, Belgium.; 19 ESCMID Study Group for Biofilms, Switzerland.; 20 Laboratory for Microbiology, Parasitology and Hygiene (LMPH), University of Antwerp, Belgium.; 21 Department of Molecular Genetics, University of Toronto, Toronto, Ontario, Canada.; 22 Department of Oral Biology, School of Dental Medicine, University at Buffalo, Buffalo, NY USA.; 23 VCU Medical Center, Richmond, VA, USA.; 24 Division of Infectious Diseases, Los Angeles Biomedical Research Institute at Harbor-UCLA Medical Center, Torrance, CA, USA.; 25 Center for Medical Mycology, Department of Dermatology, University Hospitals Cleveland Medical Center and Case Western Re-serve University, Cleveland, OH, USA.; 26 MRC Centre for Medical Mycology, Institute of Medical Sciences, University of Aberdeen, Aberdeen, UK.; 27 Biocenter - Division of Molecular Biology, Medical University Innsbruck, Innsbruck, Austria.; 28 Department of Oncology and Diagnostic Sciences, School of Dentistry; Department of Microbiology and Immunology, School of Medicine, University of Maryland, Baltimore, USA.; 29 National Infection Service, Public Health England, Mycology Reference Laboratory, Bristol, UK.; 30 Centers for Disease Control and Prevention, Atlanta, GA, USA.; 31 Department of Biology, South Texas Center for Emerging Infectious Diseases, The University of Texas at San Antonio, San Antonio, USA.; 32 Department of Microbiology and Immunology, KU Leuven, Leuven, Belgium and Clinical Department of Haematology, UZ Leuven, Leuven, Belgium.; 33 University of Wisconsin-Madison, Departments of Medicine and Medical Microbiology & Immunology, Madison, WI, USA.; 34 Department of Molecular and Cell Biology, School of Natural Sciences, University of California, Merced, Merced, USA.; 35 Departments of Pathology and Epidemiology, University of Iowa, Iowa, USA.; 36 JMI Laboratories, North Liberty, Iowa, USA.; 37 College of Medical, Veterinary and Life Sciences, University of Glasgow, UK.; 38 Institute of Microbiology, University of Lausanne and University Hospital, CH-1011 Lausanne.; 39 Institute of Microbiology, Università Cattolica del Sacro Cuore, IRCCS-Fondazione Policlinico "Agostino Gemelli", Rome, Italy.; 40 Pharmacy Dpt, University Hospitals Leuven and Clinical Pharmacology and Pharmacotherapy, Dpt. of Pharmaceutical and Pharma-cological Sciences, KU Leuven, Belgium.; 41 Center of Expertise in Mycology Radboudumc/CWZ, Radboud University Medical Center, Nijmegen, the Netherlands (omit "Nijmegen" in Radboud University Medical Center).; 42 MRC Centre for Medical Mycology, Aberdeen Fungal Group, University of Aberdeen, Foresterhill, Aberdeen, UK.; 43 KU Leuven-University of Leuven, University Hospitals Leuven, Department of General Internal Medicine, Herestraat 49, B-3000 Leuven, Belgium.; 44 Geffen School of Medicine at the University of California, Los Angeles, Divisions of Molecular Medicine & Infectious Diseases, Har-bor-UCLA Medical Center, LABioMed at Harbor-UCLA Medical Center.; 45 Department of Medical Microbiology, Amsterdam Infection and Immunity Institute, Academic Medical Center, University of Am-sterdam, Netherlands.

**Keywords:** antifungal susceptibility testing, biofilm inhibition, biofilm eradication, antibiofilm material coating, in vivo models

## Abstract

Unlike superficial fungal infections of the skin and nails, which are the most common fungal diseases in humans, invasive fungal infections carry high morbidity and mortality, particularly those associated with biofilm formation on indwelling medical devices. Therapeutic management of these complex diseases is often complicated by the rise in resistance to the commonly used antifungal agents. Therefore, the availability of accurate susceptibility testing methods for determining antifungal resistance, as well as discovery of novel antifungal and antibiofilm agents, are key priorities in medical mycology research. To direct advancements in this field, here we present an overview of the methods currently available for determining (i) the susceptibility or resistance of fungal isolates or biofilms to antifungal or antibiofilm compounds and compound combinations; (ii) the *in vivo* efficacy of antifungal and antibiofilm compounds and compound combinations; and (iii) the *in vitro* and *in vivo* performance of anti-infective coatings and materials to prevent fungal biofilm-based infections.

## INTRODUCTION

Superficial fungal infections of the skin and nails are the most common fungal infections in humans and, although rarely invasive, they can be debilitating, persistent and impose substantial treatment costs 1. In contrast, invasive fungal infections are life threatening, with a higher mortality rate per year than that by malaria, breast or prostate cancer 2. More than 90% of all reported fungal-related deaths (about one million people every year) result from species that belong to one of four genera: *Cryptococcus*, *Candida*, *Aspergillus* and *Pneumocystis* 2,3.

The most important antifungal agents (antimycotics) clinically used for systemic infections can be subdivided into four main classes: azoles, polyenes, echinocandins and pyrimidine analogues (5-fluorocytosine). In addition, allylamines (terbinafine) are frequently used against superficial fungal infections 4. The rise in azole resistance, echinocandin resistance and cross-resistance to at least 2 antifungal classes (multi-drug resistance: MDR) has been a worrisome trend, mainly in large tertiary and oncology centers. Overall, rates of antifungal resistance and MDR in *Candida* species and particularly in the emerging human pathogen *C. glabrata* are increasing 5. More concerning, the newly identified *Candida* species *C. auris* has drawn considerable attention as this uncommon species is the first globally emerging fungal pathogen exhibiting MDR to the three major classes of antifungals (azoles, echinocandins and amphotericin B and its lipid formulations) and is characterized by a strong potential for nosocomial transmission 6. In addition to *Candida*, azole resistance in *Aspergillus fumigatus* has been reported worldwide, and such resistant isolates can cause invasive infections with high mortality rates 7. Alongside the serious issues presented by classical MDR, there is another important, but less appreciated problem with our current approach to antimicrobial therapy in general. Existing antimicrobial treatments are frequently associated with therapeutic failure even against infections caused by susceptible strains due to intrinsic mechanisms that protect the micro-organisms from the antimicrobial agents, such as the formation of drug-tolerant biofilms. Microbial biofilms consist of dense layers of microorganisms surrounded by an extracellular polymer matrix, which provides biofilm-embedded microorganisms with protection against antimicrobial agents. Most bacteria and fungi exist predominantly in such organized communities in nature and, according to a recent public announcement from the US NIH, biofilms are responsible for more than 80% of human soft- and hard-tissue infections 8. Of more significance, microbial biofilms are thought to result in therapeutic failure and occurrence of resistance 9,10,11,12.

Therefore, the development of accurate susceptibility testing methods for detecting or excluding antifungal resistance, as well as discovery of novel antifungal and antibiofilm agents, are key priorities in medical mycology research. The term ‘antibiofilm agents’ relates to compounds that can inhibit biofilm formation and/or eradicate fungal cells in the biofilm.

To direct advancements in this field, we present in this review an overview of methods for use by investigators who aim to examine:

(i) susceptibility (and resistance) of fungal cultures or biofilms against antifungal or antibiofilm compounds and compound combinations;

(ii) *in vivo* efficacy of antifungal and antibiofilm compounds and compound combinations; and

(iii) *in vitro* and *in vivo* performance of anti-infective coatings and materials to prevent fungal biofilm-related infections.

Several of these topics are already covered in recent guideline-style based reviews 13,14,15,16. We refer to these reviews in the relevant sections and summarize their most important recommendations and guidelines.

**Box 1:**
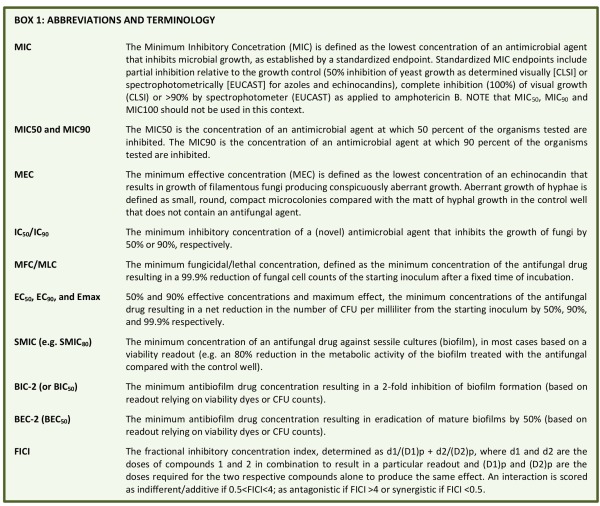
BOX 1

## METHODS FOR ANTIFUNGAL SUSCEPTIBILITY TESTING OF PLANKTONIC CULTURES

### Microdilution-broth based antifungal susceptibility testing (AFST)

Predicting therapeutic outcomes as well as guiding the antifungal drug discovery process based on AFST of pathogenic fungi remains challenging. In a recent review, Sanguinetti and Posteraro presented an overview of standard AFST methods, focusing on their advantages and disadvantages, as well as of new promising technologies and newer-generation methods (e.g. whole genome sequencing) that can predict resistance 13.

The Clinical and Laboratory Standards Institute (CLSI; formerly the National Committee for Clinical Laboratory Standards [NCCLS]) and The European Committee on Antimicrobial Susceptibility Testing (EUCAST) have developed reproducible methods for testing the activity of antifungal agents against yeasts (the CLSI M27, M44, M60 and the EUCAST E.Def 7.3 documents) and filamentous fungi (molds; the CLSI M38, M44, M51, M61 and EUCAST E.Def 9.3 documents) 17,18,19,20,21,22,23. These reference AFST methods, or their commercial counterparts such as Sensititre YeastOne (SYO, Thermo Fisher Scientific, MA, USA) rely on measuring growth of a defined fungal inoculum in a specific growth broth in the presence of different concentrations of the antifungal drug and allow the determination of the MIC (the minimum inhibitory concentration) of the drug resulting in complete or prominent growth inhibition. Clinical breakpoints have been determined by CLSI for anidulafungin, caspofungin, micafungin, fluconazole, and voriconazole against the prevalent *Candida* spp. whereas the EUCAST has set breakpoints for amphotericin B, anidulafungin, micafungin, fluconazole, itraconazole and voriconazole against the common *Candida* spp. and for amphotericin B, itraconazole, isavuconazole, posaconazole and voriconazole against the most common *Aspergillus* species 23,24. These AFST methods deliberately minimize measurement of tolerance or trailing growth (see further) because it is highly variable under different culture conditions. For drug-organism combinations for which clinical breakpoints are not available, epidemiological cutoff values are suggested based on normal ranges of susceptibility of wild-type populations 16. These should encompass the range of normal strain to strain variation within a species but exclude those organisms with known resistance mechanisms. Note that in some cases, published proposed epidemiologic cut-off values (see below) do not seem realistic. For example, for *C. auris* and fluconazole, they are so high that they fail their assignment as resistant. This has been recently addressed by the suggestion of more realistic, lower fluconazole breakpoints 25. The occurrence of resistance is often associated with a genetic difference between a susceptible and resistant isolate. Resistance may be also the result of transient and reversible adaptation 26,27.

Commonly used **MIC end-point terminologies** are: The minimum inhibitory concentration (MIC) is the lowest concentration of an antimicrobial agent that prevents or inhibits the visible growth of fungal cells, as established by a standardized endpoint. Standardized MIC endpoints include partial inhibition of growth relative to the growth observed in the control (>50% inhibition of growth as determined visually [CLSI] or spectrophotometrically [EUCAST] for azoles and echinocandins), complete inhibition (100%) of visual growth (CLSI) or >90% by spectrophotometer (EUCAST) as applied to amphotericin B 28. **NOTE that MIC_50_, MIC_90_ and MIC_100_ are NOT appropriate in this context. **The MIC50 is the concentration of an antimicrobial agent at which 50 percent of the strains of an organism tested are inhibited. The MIC90 is the concentration of an antimicrobial agent at which 90 percent of the strains of an organism tested are inhibited. The term minimum effective concentration (MEC) is used to describe the effect of echinocandin agents on filamentous fungi and is defined as the lowest concentration that results in conspicuously aberrant growth as assessed microscopically. Aberrant growth of hyphae is defined as small, round, compact microcolonies, often with swollen ends to the hyphae, compared with the matt of hyphal growth in the control well that does not contain an antifungal agent.

Interpretation of the efficacy of a given antifungal drug, is determined by the use of **clinical breakpoints (CBPs)**. The CLSI uses the term ‘breakpoint’ as ‘clinical breakpoint’ is redundant in light of the fact that breakpoints are only applicable under clinical conditions. Thus the CBPs for in vitro susceptibility testing are used to indicate those isolates that are likely to respond to treatment with a given antimicrobial agent administered using the approved dosing regimen for that agent 28. CLSI and EUCAST have established species-specific CBPs for some of the systemically active antifungal agents [CLSI 18, M60 Ed1; EUCAST E.Def 7.3 and E.Def 9.3]. The CBPs also provide information on the sensitivity of the CLSI/EUCAST methods to detect emerging resistance associated with acquired or mutational resistance mechanisms. The CBPs sort isolates into interpretive categories of susceptible (S), susceptible dose dependent (SDD; i.e. susceptibility is dose-dependent), intermediate (I), and resistant (R). The SDD category encompasses those organisms with MICs in a range that may respond to systemic therapy providing the drug levels in the blood are sufficiently high, especially relevant for fluconazole and voriconazole. For fluconazole and *Candida glabrata*, SDD is representing MICs < 32 µg/ml.. CBPs are established by taking into account microbiological (MICs and ECVs), clinical, molecular mechanisms of resistance, biochemical, pharmacokinetic and pharmacodynamic (PK/PD) data and provide the best cutoff value to predict clinical outcome for the treatment of a specific organism and antifungal agent 28.

**Epidemiologic cutoff values (ECV/ECOFF)** have been established to aid in the interpretation of MIC results when the lack of clinical data precludes the establishment of CBPs (CLSI, M57 and M59 29,30; EUCAST). As mentioned above, they are also the first for proposing BPs. It has been suggested that some CBPs may not detect known mutational resistance in different species. Because of that, an extensive effort has been undertaken to establish ECVs or the MICs/MECs that separate wild-type (WT) from non-WT strains; the latter are more likely to harbor acquired and mutational resistance mechanisms. ECVs as BP are species-and-method-specific 30 and are also useful for tracking the emergence of strains with decreased susceptibility to a given agent and therefore less likely to respond to therapy 30; ECVs can be used for the same purpose in surveillance studies 16. Epidemiologic cutoff values can also be used to identify isolates that are less likely to respond to therapy when clinical CBPs cannot be established because of the rarity of infection with unusual species of fungi. However, some PK/PD must be known about the bug/drug combination in order to determine the efficacy of a given ECV/ECOFF and account for intrinsic resistance.

**When testing the antifungal activity of a novel antifungal agent, relative to standard antimycotics, we propose to use the abbreviation ‘IC_50_ or IC_90_’, defined as the minimum inhibitory concentration of the agent or standard antimycotic that inhibits growth of the fungus (or similar readout like metabolic activity, see further) by 50% or 90%, respectively. In this way, there will not be a mix up with MIC abbreviations, which can only be used in the context of standardized AFST assays and endpoints.**

One of the major drawbacks with AFST methodologies described above is that they are time-consuming and/or have long turn-around-times. Another concern is the general subjectivity involved in reading MIC end-points and the inter-laboratory variability of MIC values, especially for methods involving visual endpoint reading and for specific antifungal drugs such as caspofungin. EUCAST testing is advantageous for yeast as an objective spectrophotometric endpoint reading is performed 23 and image analysis of disk diffusion assays 31 or measurement of optical density (OD) in broth microdilution assays can provide a normalized quantitative measure of the degree of growth inhibition in the presence of a drug relative to growth in the absence of drug. EUCAST recommends reading the MICs based on OD measurements using a wavelength of 530 nm, although other wavelengths can be used e.g. 405 nm or 450 nm.

Promising alternatives to the classical phenotypic AFST are phenotype-centered (or semi-molecular) approaches that combine a culture step with molecular analysis (i.e. by real-time PCR or matrix-assisted laser desorption ionization time-of-flight mass spectrometry (MALDI-TOF MS)) 13. However, in contrast to phenotypic methods, an important caveat of using PCR/sequencing is that it is suitable to detect resistance but not susceptibility as it can only detect resistance mechanisms that are already recognized. While the MALDI-TOF MS step of the analysis provides rapid analysis, the requirement for pre-assay cultured growth of the pathogen limits the ability to improve turn-around-times for more rapid diagnoses.

### Fungicidal activity testing

Fungistatic drugs are defined most stringently as those that inhibit growth, whereas fungicidal drugs essentially kill all (>99.9%) cells in a fungal population. Typically, fungal pathogens are efficiently eliminated by the immune system in an immunocompetent host whereas immunosuppressed individuals are highly predisposed to fungal infections. Therefore, fungicidal drugs are invaluable for this vulnerable patient population for eliminating fungal pathogens 32,33. Fungicidal drugs have an advantage over fungistatic drugs in that drug resistance is not common; however, the distinction between fungicidal and fungistatic activities could be problematic 34,35,36. For example, although caspofungin is fungicidal for most yeast species, in molds it disrupts the hyphal tips but the surviving mycelium can continue to grow in the presence of the drug 37.

The minimum fungicidal/lethal concentration (MFC/MLC) has been defined as the minimum concentration resulting in a 99.9% reduction of fungal cell counts after a fixed time of incubation. Although arbitrary, the use of 99.9% (or 3-log-unit decrease) killing of the initial inoculum is the most stringent *in vitro* criterion for determining fungicidal activity 33. In 2003, Canton and colleagues proposed a minor modification of the M27-A2 MIC procedure to allow MFC determinations based on the MIC test setup 38. A smaller inoculum of 10^4^ CFU/ml was used and the entire contents of each clear well in the MIC test were spotted onto two 90 by 15 mm Sabouraud dextrose agar plates (100 µl/plate), thereby allowing the fluid to soak into the agar. After the plate was dry, it was streaked uniformly to separate cells and remove them from the drug source. This method has been used in various studies for determination of MFC 39.

Similarly, time-kill assays can also be informative 39,40,41,42. For *Candida* spp., these are typically carried out in 10 mL RPMI 1640 inoculated with 1-5 x 10^3^ CFU/mL and concentrations of 32-, 16-, 8-, 4-, 2-, 1- and 0.5x the MICs. At predetermined time points (0-, 6-, 12-, 24-, 36- and 48 h), aliquots of 100 µl are removed from each control (drug-free) and test solution tube and then serially diluted in sterile water. A volume of 100 µL from serially diluted aliquots is placed on SDA plates to determine the number of CFU/mL after incubation at 35°C for 24 h. Using this method, Scorneaux and colleagues determined the time to reach 50%, 90%, and 99.9% reduction in the number of CFUs from the starting inoculum 42. Net change in the number of CFU per milliliter was used to determine 50% and 90% effective concentrations and maximum effect (EC_50_, EC_90_, and *E*_max_, respectively). Caspofungin was used as a fungicidal reference. Final DMSO concentrations were typically ≤1% (vol/vol) of the solution composition. Slight variations on this method use a more concentrated inoculum (10^5 ^CFU/mL) and/or smaller volume (5 mL) 43.

An alternative approach to perform time-kill studies using a BioScreen C MBR setup was implemented by Gil-Alonso and colleagues 44 where a final volume of 200 µL and an inoculum of 1x10^5^-5x10^5^ CFU/mL were used. At 0, 2, 4, 6, 8, 24, and 48 h, aliquots of 6 or 10 µL were removed from both the control and each test solution well, and were serially diluted in phosphate-buffered saline (PBS), and plated onto Sabouraud agar to determine the number of CFU per mL. For evaluating natural salt-sensitive antifungal peptides such as salivary histatins, defensins and lactoferrin, it is less clear what method is most suitable for assessing their activity, which is typically quenched in high salt media such as RPMI. In this case, kill studies are performed using yeast cells diluted in 10 mM pH 7.4 sodium phosphate buffer (NaPB) to 1 X 10^6^ CFU/mL; then mixed with different concentrations of biological peptides at 30°C for 30 and 60 min with gentle shaking. Following incubation, samples are diluted in 10 mM NaPB, then aliquots of 500 cells are spread onto YPD agar plates and incubated for 36-48 h, until colonies can be visualized 45,46.

For filamentous fungi, Espinel-Ingroff and colleagues 47 tested several conditions for optimum determination of MFC, which were subsequently adopted by other research groups 48. In these studies, the CLSI M38-A broth microdilution methods were used for MIC determination; following a 48-h incubation, 20 µL from each well without visual growth were plated on agar (Sabouraud dextrose agar) plates and MFCs were defined as the lowest drug concentration that yielded fewer than three colonies (approximately 99 to 99.5% killing).

Thus far, all the methods described for determination of fungicidal activity are dependent on the enumeration of replication-competent (i.e. culturable) cells following exposure to an antifungal agent. The inclusion of dyes validated for specific detection of either vitality or mortality in the conventional AFST MIC setups can be highly relevant for assessing the fungicidal nature of an antifungal in a direct way 33,49,50. For example, propidium iodide (PI) is a fluorescent dye that can cross only permeable membranes and fluoresces upon interaction with DNA in dead, permeable cells. Hence, PI-positivity is generally regarded as a measure of cell death. However, it is noteworthy that cells in which apoptosis is induced by antifungal compounds, although dead, are typically PI-negative 51. Of further note here is the fact that PI-positivity is not always indicating cell death as this can sometimes be restored to PI-negativity under certain conditions. In addition to fluorescent staining, tetrazolium compounds MTT (2H-Tetrazolium, 2-(4,5-dimethyl-2-thiazolyl)-3,5-diphenyl-, bromide) or XTT (2H-Tetrazolium, 2,3-bis(2-methoxy-4-nitro-5-sulfophenyl)-5-[(phenylamino)carbonyl]-hydroxide) are also commonly used to assess cell viability (with these dyes redox potential is measured as a proxy of viability) 52. Similarly, actively respiring cells convert the water-soluble XTT to a water-soluble, orange colored formazan product. However, unlike MTT, XTT does not require solubilization prior to quantitation, thereby reducing the assay time in many viability assay protocols. Similarly to the MTT-to-formazan reduction, the addition of an oxidation-reduction colorimetric indicator like Alamar blue (containing resazurin) can be used. This indicator changes from blue to the pink fluorescent resorufin in the presence of metabolically active growing cells 53. A disadvantage of resazurin can be its low level of stability when incubated for 7 days or longer (required for certain dermatophytes). As discussed further below, these dyes are most often used to assess the effect of antifungal agents against biofilms.

### Additional comments and notes with respect to MIC/MFC determinations

For studies assessing efficacy of antifungal therapy, resistance development during therapy and fungal epidemiology, it is necessary to determine clear and uniform MIC/MEC/MFC end-points according to the reference AFST methods indicated above. However, this is not necessarily the case for the identification of novel antifungal compounds during early preclinical drug discovery phases. For the latter, compliance and feasibility for high-throughput testing (either compound libraries or deletion mutant collections for investigating mode of action) are generally more important than exact end-point MIC/MEC/MFC, as long as the MIC/MFC end-points of relevant standard antimycotics are included and used for interpretation of relative activities. Deviations from reference AFST methods generally relate to different media/buffers; inclusion of serum might be most relevant when screening for fungicidal compounds that retain activity in the presence of serum; and inoculum size. The latter might need to be adjusted when using higher throughput setups based on for instance a Bioscreen apparatus.

One of the most puzzling effects that can confound MFC/MEC/MIC determination is the **paradoxical** **effect or Eagle effect **which has also been described with antibacterial agents. This effect is defined as the ability of the fungus to grow at high antifungal concentrations (above the MIC), but not at intermediate concentrations 43,54,55,56. For example, a paradoxical effect for echinocandins against various *C. albicans* isolates and against *A. fumigatus* has been demonstrated. Paradoxical growth varies in terms of media, species, strain and type of echinocandin and is also of specific concern in the biofilm field (see further). Rueda and coworkers demonstrated that paradoxical growth of *C. albicans* is associated with multiple cell wall rearrangements and reduced virulence 57. It is important to note however, that observed *in vitro* paradoxical growth does not necessarily indicate lack of response to the antifungal drug *in vivo* 58. Therefore, the clinical implications of fungal adaptation against antifungal drugs, which might be linked to reduced virulence, still remain to be elucidated. In addition, MIC/MEC deviations can be induced by the presence of active volatile compounds in neighboring wells of a microtiter plate. By screening a large collection of essential oils it was shown that ‘paradoxical like phenotypes’ were caused by small volatile molecules released from adjacent wells. This observation led to the development of a quantitative method to evaluate the activity of volatile molecules in the vicinity 59.

Another unclear issue has been the description of drug responses as tolerance or trailing growth, which has often been measured using modifications of MIC assays and is particularly relevant for azole and echinocandin (both *Candida *spp. and filamentous fungi) activity against *Candida *spp 60,61,62,63. Tolerance may be perceived as a subpopulation effect that is due to slow growth of a subpopulation of cells in a manner that is generally drug-concentration independent within the range of concentrations studied 64. Of note, standard MIC values are determined following at 24h growth period; however, tolerance may become evident after 48h of growth in the presence of the drug. Tolerance can also be detected by agar disk diffusion assays 31,64. Recently, additional definitions have been proposed in this context, namely fraction of growth (FoG) inside the zone of inhibition and the Supra-MIC growth (SMG) in drug concentrations above the MIC. The FoG and SMG measurements correlate with one another and are clearly distinct from measurements of the MIC or the radius of the zone of inhibition 31,64.

## METHODS FOR ANTIFUNGAL SUSCEPTIBILITY TESTING OF BIOFILMS

Microbial biofilms consist of dense layers of microorganisms surrounded by an extracellular polymer matrix, thereby protecting the microbes from the action of antimicrobial agents. In addition, dormant persister cells have been proposed to make up a small proportion of some microbial cultures (either a biofilm or free-living culture) that can withstand the action of high doses of most antimicrobial agents and may facilitate the recurrence of microbial infections after treatment ceases 65,66. In medical mycology, the appearance of persister cells is often referred to as ‘heteroresistance’, as the cells are not resistant in the classical sense. Rather, they transiently acquire the ability to survive and grow at normal growth rate under conditions that inhibit growth of the majority of the isogenic susceptible population 67,68,69,70. Heteroresistance is defined as a small proportion of the population (usually <1%) that is able to grow in the presence of the drug. This is different from tolerance or trailing growth, where many cells in the population (>1%) are able to grow, albeit slowly, in the presence of the drug.

The majority of *C. albicans* infections are associated with biofilm formation 71,72,73,74,75. *A. fumigatus* is the most important airborne human fungal pathogenic mold and the number of chronic *A. fumigatus* infections is constantly increasing in patients suffering from respiratory tract diseases. Until recently, most studies undertaken to understand *Aspergillus* physiology and virulence were performed under free-living conditions. However, in all *Aspergillus* infections, *A. fumigatus* grows as a colony characterized by multicellular and multilayered hyphae that are in some cases embedded in an extracellular matrix (ECM). This type of growth is generally consistent with the definition of a biofilm 76. Therefore, it is becoming increasingly important to also assess the activity of current antimycotics and novel antifungal compounds against biofilms.

Several methods for fungal susceptibility testing under biofilm conditions for determination of sessile MICs (SMICs) have been developed which in principle are based on methods for planktonic culture testing 13,71,76,77. The most commonly used method is based on a static model using 96-well microtiter plates 78,79, thereby quantifying microbial biomass or metabolic activity, using compounds such as crystal violet (CV), 2,3-bis (2-methoxy-4-nitro-5-sulfophenyl)-5-[(phenylamino)carbonyl]-2H-tetra- zolium hydroxide reduction (XTT), the more reliable fluorescein diacetate (DFA), or resazurin 80. Though, caution should be exercised in the particular assay employed, as each assay has its pros and cons depending on the question being addressed, e.g. XTT should only be used to compare the effect of an active within a specific strain and not to compare different strains 81. Propidium iodide (PI), described above, is also used to assess the degree of killing of biofilm cells. Recently, a biofilm model for *A. fumigatus *was reported, in which conidia and hyphae are trapped in an agar layer with measurement of metabolic activity by the XTT assay 82. A newer high-throughput approach grows biofilms with shaking in 96-well and 384-well plates in the presence of the compound of interest, and uses optical density at 600nm (although other wavelengths can be used) as a readout of the biofilm remaining during or after exposure to the compound 83. As indicated by the authors, the optical density of the biofilm correlates with the number of viable cells in the biofilm, and thus is an accurate read-out of biofilm formation in the presence of the compound of interest. This method returns consistent results with less labor and can be performed in high-throughput. A more labor-intensive approach for analysis of *C. albicans* biofilms involves determining the number of CFUs recovered from treated biofilms, which involves harvesting the biofilm by scraping, homogenizing and plating homogentaes on agar media for colony enumeration. Homogenization can be done by vortexing (potentially in the presence of 1% Triton) or sonication. However, these procedures must ensure that all biofilm cells are harvested and individually separated without affecting the viability of the biofilm cells, three conditions that can be difficult to achieve. Another limitation of cultivation-based assays lies in the fact that biofilms contain hyphae which might bias CFU counts. Ideally, measurements should rely on nucleus counting which can be performed using qPCR. In theory, it is recommended to use at least two assays that rely on different methods or dyes as a readout.

While static biofilm models in 96-well plates are used most frequently, continuous flow models that better mimic *in vivo* situations can be used to assess *C. albicans* biofilm formation and inhibition 84,85. Unlike static biofilms, *C. albicans* cells under constant laminar flow undergo continuous detachment and seeding that may be more representative of the development of *in vivo* biofilms 86. A particularly relevant example is the study of oral biofilms, which form in the presence of salivary flow. Most notably, it was shown that biofilm cell detachment rates are an important predictor of ultimate biofilm mass under flow 86. However, whether these flow models are indeed *better* will depend on the pathogenesis of the infection under study.

Microscale technologies, such as microfluidics provide a more revolutionary approach to study biofilm formation in dynamic environments. By enabling control and manipulation of physical and chemical conditions, these technologies can better mimic microbial habitats in terms of fluid flow and nutrient sources 87. Gulati and coworkers recently described a protocol to study biofilm formation in real-time using an automated microfluidic device under laminar flow conditions 88. This protocol enabled the observation of biofilms in real-time, using customizable conditions that mimic those of the host, e.g., conditions encountered in vascular catheters. This protocol can be used to assess the biofilm defects of genetic mutants as well as the inhibitory effects of antimicrobial agents on biofilm development in real-time. Most recently a novel technique consisting of nano-scale culture of microbial biofilms on a microarray platform was developed whereby thousands of microbial biofilms, each with a volume of approximately 30-50 nanolitres are simultaneously formed on a standard microscope slide. Despite a 2000-fold miniaturization compared to microtiter plates, the resulting nanobiofilms display similar structural and phenotypic properties. This technique platform can significantly speed up biofilm susceptibility testing and allows for true high-throughput screening in search for new anti-biofilm drugs 89,90,91.

In addition to the SMIC, the BIC-2 (BIC_50_), is the minimum compound concentration resulting in a 2-fold *inhibition* of biofilm formation. Similarly, the BEC-2 (BEC_50_) is the minimum compound concentration resulting in a 2-fold *eradication* of mature biofilms 92,93. In practice, to assess biofilm inhibition, compounds are included during the biofilm growth phase. To assess biofilm eradication however, biofilms are grown for 24h after which the compounds are added and biofilms are additionally incubated for another 24h in the presence of the compounds. In that respect, BEC determination is most relevant for compounds that can kill biofilm cells, whereas BIC determination is relevant for compounds that might only inhibit adhesion without impacting viability of biofilm cells. Hence, biofilm inhibiting compounds can be very relevant for the design of antibiofilm material coatings (see further), whereas biofilm eradicating compounds can be used to design curative antibiofilm therapy. With regard to terminology, SMIC_20/50/80_ can also be used (see before): SMIC_80_ has been defined previously as an 80% reduction in the metabolic activity of the biofilm treated with the antifungal compared to that of untreated biofilms 60. In general, SMICs can be up to 1000-fold higher than the corresponding MICs for a particular antifungal agent 94. Among the different mechanisms that may be responsible for this intrinsic tolerance of *Candida* species biofilms are: the high density of cells within the biofilm; nutrient limitation within the biofilm; effects of the biofilm matrix; antifungal resistance gene expression; and the increase of sterols in biofilm cell membranes 95,96.

Although it is clear that a wide range of (standardized) techniques is available to determine the activity of compounds against biofilms, there is currently little evidence that implementing these biofilm-based assays in the clinical microbiology laboratory lead to better treatment outcomes 97.

### Synergistic antifungal/antibiofilm drug combination testing

In addition to screening for novel antifungal compounds, combination therapy is considered a potential alternative strategy for treating invasive fungal infections 98,99. In general, the main objective of combination therapy is to achieve a synergistic interaction between two compounds, thereby increasing their activity and reducing potential toxic effects of each compound. Apart from combining two antifungal (or antibiofilm) agents that are characterized by different mode of actions that can synergize each other, another option is to combine an antimycotic with a non-antifungal potentiator. An increasing number of studies document the synergistic action of such antifungal-potentiator combinations, with the potentiators being (lasso)peptides like antifungal tyrocidines, humidimycin, or plant defensins, or repurposed compounds like toremiphene or artesunate 92,100,101,102,103,104,105,106. Repurposing of known drugs, i.e. finding novel therapeutic indications for existing drugs, is favorable from an economic perspective, as these molecules are often FDA-approved and have a known (and often safe) toxicity profile and dosing regimens are known. Furthermore, the cost of performing new clinical trials with existing drugs with possibly reformulating the drug are likely to be far lower than the costs of developing a new drug.

An interaction between two compounds is defined as synergistic when the combined effect of the two compounds is greater than the sum of their separate effects at the same doses. Synergistic efficacy can be quantified *in vitro *using checkerboard assays, where two-fold dilution series of one compound are combined with two-fold dilution series of the other compound and scored for the read-out of interest (e.g. growth inhibition, killing, biofilm inhibition, biofilm eradication). The fractional inhibitory concentration index (FICI) is determined as d1/(D1)p + d2/(D2)p, where d1 and d2 are the doses of compounds 1 and 2 in combination to result in, for example, 50% inhibition of biofilm formation and (D1)p and (D2)p are the doses required for the two respective compounds alone to produce the same effect. An interaction is scored as indifferent/additive if 0.5<FICI<4; as antagonistic if FICI >4 or synergistic if FICI <0.5 107.

Although the FICI is most frequently used to define or describe drug interactions, it has some important disadvantages when used for drugs against filamentous fungi. This includes observer bias in the determination of the MIC and lack of agreement on the endpoints (MIC-0, MIC-1, or MIC-2 [≥95, ≥75, and ≥50% growth inhibition, respectively]) when studying drug combinations 108. Moreover, when one compound strongly potentiates the other but the reverse is not the case, the FICI value will not reach values below 0.5. Synergy then is not concluded, whereas there certainly can be a very relevant reduction in concentration of major antifungal agents, with concomitant reduction of toxic effects in (prolonged) treatment.

## METHODS FOR TESTING OF MATERIALS AND COATINGS THAT RESIST FUNGAL BIOFILM FORMATION

Fungal adhesion and subsequent biofilm formation on biomedical implants and devices are a major cause of biofilm-associated infections. Because treatment of such infections is very difficult (see previous section), emphasis has shifted to the prevention of these infections by the design of antimicrobial coatings for biomedical devices. In general, to prevent microbial biofilm formation, coatings rely on either of three principles: they can act via release of antimicrobial agents, via coating of antimicrobial agents (resulting in contact-cidal activity) or via coating of anti-adhesive agents that are not antimicrobial. In a recent review by Sjollema and colleagues 14, 15 methods or groups of methods to assess *in vitro* performance of these three types of antimicrobial coatings were discussed. To evaluate the efficacy of one of the three antimicrobial designs independently, no single method could be determined to be "one for all" with all having different merits for different antimicrobial designs.

Many antimicrobial designs in clinical practice and described in literature are based on the slow or fast release of antimicrobials 109. By far the most applied methods to evaluate the effect of antimicrobial release on microbial growth inhibition are "agar zone of inhibition" methods 110,111, very similar to disk diffusion susceptibility tests as described by the CLSI standard for yeasts (CLSI M44-A). In these methods, a plate with nutrient agar media is inoculated with microorganisms and a test sample is subsequently placed with the antimicrobial side on the agar. Following an incubation period during which released antimicrobials from the test sample diffuse into the agar, a zone of growth inhibition is formed the diameter of which is indicative of the level of susceptibility of the microorganisms to the antimicrobials and the amount released.

Although this method is effective, simple to execute and available in most microbiology labs, it does not quantify the antimicrobial efficacy 112. For quantification of efficacy of surface designs based on release of antimicrobials, various assays are described which are mainly categorized as "suspension methods". In these assays, a known inoculum of microorganisms, suspended in a nutrient medium, is exposed to a test sample and incubated for a set period of time (usually 1 to 2 days, but for moulds a longer period) 113. Following incubation a sample of the suspension is taken and the number of surviving microorganisms is enumerated (for bacteria and yeasts often by CFU, for fungi quantitative colorimetric XTT assays are more appropriate 114).

A variation of this suspension method is recommended in situations where a clinical scenario is characterized by a very small volume/area ratio, such as in the narrow extraluminal space between a urinary catheter and the urethral epithelium, where released antimicrobials accumulate rapidly. Typically, in "high area to volume tests" a small volume of a microbial suspension is incubated, sandwiched between a thin cover sheet and the test sample, therewith stimulating intimate contact between the microorganisms and the sample 115. Typical examples are the JIS-Z 2801 116 and "all in one" plating systems (e.g. for yeasts and fungi Petrifilm® Yeast and Mold all-in-one-plating systems, 3M, St. Paul, MN, USA) 117,118. Because the intimate contact between microorganisms and sample is established in these type of "high area to volume tests" they are often applied in evaluating contact killing designs 119.

Intimate contact is also established in "adhesion based assays" where microbial cells suspended in a low nutrient suspension are allowed to settle. Non-adhering organisms are subsequently removed by washing and adhering cells are counted microscopically or cultured following sonication 120. Flow systems, a special adhesion-based assay in which microorganisms are exposed to a sample surface from a flowing suspension, mimic flow in clinical environments such as in the intraluminal area of a urinary catheter or the extraluminal area in intravenous catheters 121. Another advantage of flow systems is that passage of samples by liquid-air interfaces and sonication applied as critical steps before assessment, can be circumvented in case of the application of real time microscopy since non-adhering microorganisms are continuously flushed away 118,122. Adhesion-based methods are preferred in the evaluation of non-adhesive surface designs.

In order to study the efficacy of antimicrobial designs in preventing biofilm formation, biofilm methods are applied 123,124. These methods resemble the "adhesion based" methods in that the process begins with an adhesion step in a low nutrient environment and over an extended incubation period, adhering microorganisms form a biofilm. In order to prevent microbial growth in the suspension, prior to the incubation step, non-adherent cells are removed by washing. The biofilm methods can be adapted to evaluate all types of antimicrobial designs.

Although several methods have been applied in research laboratories and industry, minimal guidance is provided on how to discriminate release, contact killing and non-adhesive systems. For instance, in the development of novel surface microbicidal coatings, it is important to accurately assess whether antimicrobial activity arises from direct contact or from inadvertently released compounds or components. Such differentiation may be key for successful translation to a product, as medical devices with non-releasing surface coatings are considered "pure" medical devices while release-systems are considered to be so-called combination products. Combination products entail a different regulatory pathway, i.e. the whole entire pharmaceutical activity of the released therapeutic product has to be considered requiring a battery of *in vitro* testing and extensive toxicology and safety assessment 125. Therefore, there is a need for simple industry standards that allow discrimination between the various antimicrobial designs and in particular incorporate a presently lacking standard test for adhesion under flow conditions that resemble the flow occurring in various clinical applications.

## METHODS FOR MONITORING *IN VIVO* PERFORMANCE OF ANTIFUNGAL AND ANTIBIOFILM DRUGS

Compounds that perform well under *in vitro* testing conditions and show no or low toxicity should be validated under more physiologically relevant conditions. To this end, different animal model systems have been developed (see table 1) to evaluate efficacy of antimicrobial agents *in vivo*. However, in an effort to minimize the use of animals, three-dimensional organoid (*ex vivo*) tissue systems have been developed to screen for new drug candidates. This *in vivo* or *ex vivo* evaluation of candidate molecules is essential as many compounds that are very potent *in vitro*, fail the *in vivo* test 126. Moreover, such testing is required in view of toxicity assessment and progression to clinical application.

**Table 1:** Overview of *in vivo* models for assessing efficacy of antifungal drugs or treatments [references]..

**Animal species**	**Type of infection**	**Candida sp.**	**Aspergillus sp.**	**Cryptococcus sp.**	**Other fungi**
*Galleria melonella* (greater wax moth)		127,128,129,130,131,132,133,134,135,136,137,138,139	133,140,141,142,143,144	145,146,147,148,149	150,151,152,153,154,155
*Bombyx mori* (silkworm)		156	157	158,159,160	
*Caenorhabditis elegans*		100,136,161,162,163,164,165,166,167,168,169,170,171,172,173		166,174,175	176
*Drosophila melanogaster*		177,178	179		180
*Danio rerio* Zebrafish larvae		165		145	181,182
Mice	systemic	183,184,185,186,187,188	189	190	191,192
Mice	oropharyngeal	184,193,194,195,196,197			
Mice	vaginal	198,199,200,201,202			
Mice	Biofilm, including dentures/keratitis	203,204,205,206,207,208			
Mice	intratracheal/lung/ central nervous system		209,210,211,212,213	147,214,215,216,217	218
Mice	Cutaneous	219	220		180
Rats	Biofilm	93,101,221,222,223,224,225,226			
Guinea pigs		227	228,229	230	231

Human three-dimensional organoid tissue culture approaches or *ex vivo* human tissue, that both can be combined with specific immune cells have been developed, mainly in the cancer field 232. Recently a method was developed to mimic the large intestine where colonic organoids are generated from differentiated human embryonic stem cells or induced pluripotent stem cells 233,234. This and similar approaches will likely be used in the future for disease modeling and drug discovery 234. To study *C. albicans* infections, a human skin model was developed which can be used to test the effect of novel antibiofilm compounds, mainly for their effect on inhibition of *C. albicans* adhesion 235. Similarly, an organotypic model of the human bronchiole was developed for testing host pathogen and three-way host, fungal and bacterial pathogen communication and immune response 236. This model can also be used to test the effect of antimicrobial compounds during host-pathogen interaction.

The most relevant models however, remain animal model systems as these allow for the study of pharmacodynamics and pharmacokinetics of novel compounds (see table 1). In general vertebrate model systems are preferable as they are phylogenetically closer to humans; however, several lower, non-vertebrate, models have been optimized for virulence evaluation as well as for drug screening 15,237. Among these model systems, the most used are *Caenorhabditis elegans* 161,238, *Drosophilla melanogaster* 177,179,239, zebrafish larvae (*Danio rerio*) 140,141, the silk worm *Bombyx mori* 145,240, the heat tolerant two-spotted cricket 241 and the larvae of the greater wax moth *Galleria mellonella *140,242,243. The main advantage of the latter two is that these organisms can be maintained and grown at 35 °C to 37 °C, approximately the same as the human body temperature.

Developing a relevant model system requires considerable effort and time, and some examples of how to set up *Aspergillosis* model systems for drug discovery are described by Paulussen *et al.* 244 and Lewis and Verweij 245. When performing animal experiments to test the efficacy of drugs it is important to clearly define the endpoint in terms of organs or tissues positive for fungal presence at a particular time point during / after initiation of treatment, since the niche in the host might influence the outcome. A typical example is the pH dependent virulence of *C. albicans*, where a strain may not be virulent systemically but is virulent vaginally or vice versa 246,247,248. Recently, it was shown that fitness cost, often associated with drug resistance, is also niche-dependent as fluconazole-resistant isolates were outcompeted in a gastrointestinal colonization model and not in a systemic infection model system 249.

Vertebrate model systems in use for testing novel antifungal drugs, are primarily based on those developed to investigate virulence mechanisms by fungal pathogens 250. These model systems can be classified as methods to test therapeutic efficacy against superficial (skin, nails), mucosal (oral, vaginal), gastrointestinal, lung or systemic infections. In order to mimic the population at risk for certain types of fungal infections, in many cases animals are rendered immunosuppressed, which may influence the efficacy of a specific antifungal or compound used. Sex of the animals and route of administration of the pathogens will also determine the type and outcome of the infection. For opportunistic pathogens, such as *Candida *spp., various infection sites can be used that may either result in a systemic infection (intravenous, intraperitoneal, infections) or in local infections (oral cavity, gut, vagina or skin infections) [reviewed in 250]. In addition to testing the effect of antifungal drugs, animal model systems are also used to investigate the pharmacokinetic, tissue distribution and stability of antimicrobial drugs 251,252.

### Superficial infection models

Different animal model systems have also been developed to test the efficacy of antifungals against dermatophytes and other fungi, such as *C. albicans* that can cause skin infections 253,254,255. The guinea pig is the standard model system for these type of skin, hair and nail infections but mouse model systems have also developed such as for *C. albicans* 219,256 and *A. fumigatus* 220,257.

Experimental induction of **dermatophytosis** in laboratory animals has been performed for decades and early publications date from the 1960s 258. Guinea pigs have always been one of the target animals as they tend to be predisposed for dermal fungal infections and consequently, artificial infection is easy and does not require any kind of preconditioning or immunosuppressant treatment 259. To establish a dermatophyte infection in guinea pigs, different methods can be employed. When the model was first being used and described, it was believed that occlusion of the inoculated area was essential to induce an experimental infection 260. In the more recent publications, however, occlusion is only rarely applied and was even demonstrated not to offer any advantages on the establishment of a skin infection 253. General agreement exists on the necessity of prior trauma of the skin, before applying the inoculum. Skin damage can be achieved by using a manual razor or tape stripping, or scarification with a pumice stone, sandpaper or other methods. The most frequently used fungal species to induce experimental dermatophytosis are *Microsporum canis* and *Trichophyton mentagrophytes*, both zoophilic species but also common causative agents of human infections 259. The inoculum size usually varies between 1x10^5^ to 1x10^7^ CFU/ml 253,259. In contrast with dermatophyte infections in humans, animals show spontaneous clearance of this disease with formation of a partial immunity against reinfection 261. Methods to evaluate antifungal efficacy involve microscopic or culture examination of skin scrapings, skin biopsies, scales and/or hair samples, and histopathology of skin biopsies, while other groups use a scoring system 262,263.

**Onychomycoses** are fungal nail infections responsible for 50% of all nail dystrophies and mostly caused by dermatophytes, of which *Trichophyton rubrum* and *T. mentagrophytes* are the most important ones. Most of the studies on the biological activity of topical antifungals are based on *in vitro* models, since the existing rabbit 264 and guinea-pig 265 models are extremely time-consuming, labour-intensive and expensive.

### Mucosal infection models

**Vulvovaginal candidiasis (VVC)** is one of the models used to study mucosal infection with *Candida *spp., especially *C. albicans*. Vaginal candidiasis has been studied in mice, rats, guinea pigs and rabbits with mice and rats appearing the more sensitive species and therefore most commonly used for this type of infection. More so, the rat model offers more accurate data compared to the mouse model and is therefore the better model to study antifungal efficacy. However, like most mucosal models of candidiasis, the establishment of VVC requires predisposition of the animal, which is usually obtained by ovariectomy and hormone injections to maintain permanent oestrus, although immunosuppression or antibiotic treatment have also been employed 266. For infection, a cell suspension is directly introduced into the vagina, and disease development is assessed based on microbial recovery (CFUs) from vaginal swabs. However, histopathology and immunological analysis can also be performed with this model 231,267,268.

For studying oral candidiasis, infections are induced by placing a swab impregnated with fungal cells for a specific period of time under the tongue or rodents, and antimicrobial efficacy is assessed based on the level of microbial recovery and histopathology of infected tissue 269. Recently a model was described to induce concurrent oral and vaginal mucosal *Candida* infections 270.

### Lung infection models

For airborne infections (*Cryptococcus *spp., *Aspergillus *spp., dimorphic fungi) the model of choice is the mouse lung infection model where on average 10^4^ fungal cells are administered through inhalation, intranasal or intratracheal administration 271. Antifungal treatment is generally initiated 24-48 hours post-infection, although starting at earlier time points (after several hours or 1 day) is also reported. The current standard to treat such infections is amphotericin B (AmB) and this can be used as a control (0.5mg/kg/day) 272. This resembles the current treatment of such infections in humans where a combination therapy consisting of AmB and 5-fluoro cytosine is used to treat *Cryptococcus* central nervous system infection 273 and voriconazole is used to treat aspergillosis 274. Apart from pulmonary infections, *Aspergillus sp.* can cause a wide variety of infections and for several of them, relevant animal model systems have been developed. Some examples include the development of a sinusitis infection model 209, a rat model to study asthma 275 and a guinea pig endophthalmitis model 228. In addition to *Cryptococcus* and *Aspergillus*, model systems for other environmental fungi have also been developed such as a fusariosis model system 276, a sporotrichosis model system 277, a mucorales model system 278,279 and a model system for *Scedosporium* 280 (see Table 1). Efficacy of treatment of these fungal infections is often performed by non-culture based methods, such as qPCR to determine organ fungal burden 281.

### Gastrointestinal infection models

Gastrointestinal infections are induced by oral gavage of fungal cells. To establish a colonization, first the endogenous microbiota is partially cleared by administration of antibiotics, followed by a single oral gavage with *C. albicans* and stool samples are analyzed to determine colonization. Following euthanization segments of the gastrointestinal tract can be harvested and analyzed for CFUs (or BLI, see further) 282.

Gastrointestinal colonization model systems can also be used to induce a systemic infection which is reflective of one of the natural routes of a systemic infection in humans 283. Recently a model was described where an oral infection was initially established in mice administered tetracycline and prednisolone, which was followed by an infection of the small intestine and subsequent systemic infection 284. Specific diet-based mouse models for systemic infection via the gastrointestinal tract have been developed 285. Treatment of the animals with either fluconazole or echinocandins resulted in a significantly reduced organ microbial burden, and therefore this model would be suitable to study the efficacy of drugs in inhibiting of dissemination from the gastrointestinal tract. Interestingly, gastrointestinal tract infections have also been shown to lead to echinocandin resistance in *C. glabrata* when used in a mouse model of colonization and systemic dissemination 286. Therefore, these models are also ideal to test novel antifungal compounds or combinations of compounds*. C. albicans* is normally not part of the microbiota of the gastrointestinal tract in mice and therefore, it is not an animal model that can be colonized by *C. albicans* under natural conditions. However, the group of Lois Hoyer showed that piglets are naturally colonized by *C. albicans* and successfully used this model for gastrointestinal infections, although therapeutic studies have not yet been performed in this model 287.

### Systemic infections

Systemic infections with *C. albicans* are mostly induced using the mouse tail vein infection model system, where between 10^4^ and 10^6^ *Candida* cells are injected in the lateral tail vein 288,289. Whereas for normal virulence assays the inbred lines BALB/c or C57bl/6 are mainly used, for antifungal drug screening, outbred lines such as ICR or CD-1 should be used 183,290.

Antifungal therapy can be administered prior to infection, on day of infection, or at later time points post-infection and depending on the compound, administration could be via the oral, intraperitoneal or intravenous route. It is important here to iterate that the inoculum dose is critical; for example, the difference between the most virulent and the least virulent strain of *C. albicans* can be compensated for by adding a 10 fold excess of the latter as the inoculum, in a systemic mouse model of infection 291. Survival curves, animal weight and/or tissue burdens are determined as a measure for the efficacy of the drugs 289. The tail vein model system has been used to test the efficacy of different antifungal drugs using different *Candida* ssp. as well as other fungal pathogens 292,293,294.

### Pharmacokinetics

When discussing dosing in animal models, it is important to consider that elimination of fungal pathogens in animals and humans may vary greatly. Hence, comparing activities of two agents in an animal model using doses extrapolated from those used in humans based on weight may be misleading, as the antifungal exposure may not correspond to the one achieved in humans. This is an important caveat when comparing drugs to each other unless it is documented that the bioavailability is comparable. A case in point, comparing the activity of echinocandins in rodent model systems will not fully represent the activity in humans because the clearance and the volume of distribution is different between e.g. rats versus primates/humans. In fact, the clearance of all three echinocandins is approximately 6-fold higher in rats than in humans 295,296,297,298; specifically, caspofungin and anidulafungin have a volume of distribution that is 2- to 3-fold higher, compared to that in humans, and in micafungin this is only slightly higher in rats compared to humans 297. This difference is probably the reason why in rats higher concentrations of micafungin were required compared to caspofungin and anidulafungin to clear *C. albicans* biofilms in a subcutaneous biofilm model system 223. Also, voriconazole is rapidly metabolized in mice and rats due to autoinduction of cytochrome P450 299. Thus, rodents require much higher doses of voriconazole than human to achieve similar drug exposure.

Kinetic experiments to monitor the efficacy of drug treatment over time require large numbers of animals when CFU counting is used to analyze the effect of the drugs or survival. To overcome this issue, various imaging approaches including fluorescence and bioluminescence have been developed as they allow longitudinal experiments in single animals. For this purpose, *C. albicans* and *C. glabrata* strains have been engineered to express luciferases in order to perform bioluminescence imaging 300. *Gaussia princeps* and firefly luciferases have been codon-optimized for use in *C. albicans* and were used to monitor superficial infections such as vaginal and oropharyngeal infections 198,301,302, subcutaneous infections 303 and systemic infections 304. Recently, a red-shifted firefly luciferase was optimized for bioluminescence imaging 184. However, a limitation of the bioluminescence approach is that the presence of oxygen is required for the generation of light by luciferase. Thus, this approach will underestimate the fungal burden in established fungal lesions in which there is hypoxia. In addition to the bioluminescence approaches, fluorescence-based approaches were also developed such as the bronchoscopic fibered confocal fluorescence microscopy, which was used to assess pulmonary *Aspergillus* and *Cryptococcus* infections in live animals. This approach also allows longitudinal imaging of the same animal 305. Computed tomography and magnetic resonance imaging was also used to assess invasive pulmonary aspergillosis over time in a single animal 306. Correct diagnosis of fungal infections can be problematic, however recently imaging-based tools were developed to accurately diagnose infections such as fungal keratitis. Based on the structure of caspofungin a chemical probe exhibiting high affinity to *Aspergillus *and possibly also other fungal pathogens was developed which could serve as a basis for development of an imaging system 307.

### Biofilm models

Biofilms are notoriously difficult to treat and eradicate and therefore, several biofilm model systems have been developed and used to test the efficacy of antibiofilm molecules 308,309. The first model was a rabbit model of *C. albicans* biofilm-associated catheter infection using a catheter-lock system to determine the efficacy of antifungal treatment 310,311. In the same year, Andes *et al.* developed a rat central venous catheter (CVC) model which was also used to test the efficacy of antifungals 72,193,203,312,313. Although the CVC model has the advantage of closely mimicking the clinical situation in a patient, the disadvantage is that it is technically demanding and that only one catheter can be placed in an animal. To overcome this limitation a subcutaneous catheter rat model system was developed where in a single animal up to nine catheter fragments can be implanted. To develop a biofilm infection, catheter fragments are inoculated with *C. albicans* *in vitro* prior to implantation in the animals and biofilm is allowed to develop 314. This model system was used to test the efficacy of mono or combination treatment with antibiofilm molecules 221,222,223 and was also adapted for use with *C. glabrata* 224. Another rat model was developed to study *Candida*-associated denture stomatitis, a prevalent oral condition stemming from the ability of *Candida* to adhere to denture material and form biofilms with commensal oral bacteria 204,315,316.

Apart from traditional antifungal compounds, there is also a growing interest in the use of probiotic bacteria as agents against *C. albicans*. In a recent study, the antifungal activity of clinical isolates of *Lactobacillus* strains were tested against *C. albicans* biofilms *in vitro* and it was shown that certain species of lactobacilli had an effect on *C. albicans* morphogenesis and biofilm formation 317,318. Using the *G. mellonella* model system the same group showed that these probiotic bacteria reduce filamentation by *C. albicans* and also stimulate the host innate immune system, thereby protecting the host against *C. albicans* infections 319.

In any of the above models, it is important to consider that fungal organisms often have metabolic and phenotypic distinctions in the context of biofilms as compared to non-biofilm settings. Parameters that may affect antifungal susceptibility in experimental biofilm models *in vitro *or *in vivo *include the effects of quorum sensing 320, impact of pH signaling in abscess biofilms 321, metallic stress as may be amplified in context of devices 322, influences of host factors including immune effectors 323,324 and other micro-environmental factors. Thus, a key goal of antifungal susceptibility modeling may be evolving to specialized assay conditions that most accurately correlate with or predict outcomes in specific clinical conditions, rather than universal testing systems.

## METHODS FOR MONITORING *IN VIVO* PERFORMANCE OF ANTI-INFECTIVE MATERIALS

*In vivo* model systems have also been developed to test the efficacy of coated implant material against fungal and bacterial pathogens. A modification of the subcutaneous catheter biofilm model described above was used to test the efficacy of titanium discs coated with caspofungin, vancomycin or other small antimicrobial compounds. In this model, small (0.5 cm diameter) titanium discs that were coated with the antimicrobial compound through covalent attachment using a linker or only the linker as the negative control, were implanted subcutaneously in the back of immunosuppressed Wistar rats. The following day, animals were infected with either *C. albicans* or *S. aureus* cells in the area of the implanted discs and microbial adhesion on the discs were determined after 2 days (bacterial cells) or 4 days (fungal cells). Using this *in vivo* model, caspofungin- or vancomycin-coated discs harbored significantly lower numbers of adherent fungal and bacterial cells, respectively 325.

## CONCLUSIONS AND FUTURE PERSPECITVES

The increasing impact of fungal infections on society is mainly due to the increasing population of patients at risk, as well as the rather limited armory of antifungal agents and resistance development. Therefore, there is a critical need for the identification and development of new antifungal agents, or antifungal combination therapies, particularly those that are also active against fungal biofilms and do not suffer from resistance development. To accomplish this, a set of standardized, simple guidelines describing the appropriate methods to assess the performance of novel antifungal and/or antibiofilm agents is warranted. Consistent with the recent emphasis on preventing infections, many efforts are focused on developing antibiofilm coatings for medical devices such as catheters and implants. Thus, standardized testing of such materials is of considerable importance.

To that end, in this review we aimed to provide a compilation of general methodologies and definitions for assessment of susceptibility of fungal species to antifungal agents, when grown as planktonic cultures or as biofilms focusing on the standardized AFST guidelines. However, more recent developments and technologies in susceptibility testing designed to better mimic *in vivo* conditions with respect to flow and nutrient conditions are also discussed. These methods are still in their infancy and new guidelines for their use will need to be established in the future.

There is a plethora of abbreviations to quantify an antifungal or antibiofilm effect (see box 1), and various abbreviations have been used in different contexts. However, we want to emphasize that the MIC can only be used in the context of standardized AFST assays and endpoints, mostly reference or methods comparable to those methods. **When testing the antifungal activity of a novel antifungal agent, relative to standard antimycotics, we propose to use the abbreviation ‘IC_50_ or IC_90_’, defined as the minimum inhibitory concentration of the agent or standard antimycotic that inhibits growth of the fungus (or similar readout like metabolic activity) by 50% or 90%, respectively. MIC50 or MIC90 should only be used in an epidemiology context. **For clarity, we recommend to always indicate the complete definition of an abbreviation in any report or article.
